# Assessments of working group effectiveness in the planning of the New Jersey Kids Study: An applied mixed-methods study on the science of team science

**DOI:** 10.1017/cts.2024.578

**Published:** 2024-10-14

**Authors:** Ralph A. Gigliotti, Melissa Weidner, Michelle Jansen, Patricia Greenberg, Gloria Bachmann, Maria Gloria Dominguez-Bello, Veenat Parmar, Reynold A. Panettieri, Nancy Reilly, Charletta A. Ayers, Barrie Cohen, Lisa K. Denzin, Cecile A. Feldman, Nancy Fiedler, Manuel E. Jimenez, Robert J. Laumbach, Steven K. Malin, Natale Mazzaferro, Shilpa Pai, Todd Rosen, Lisa Rossman-Murphy, Jessica E. Salvatore, Kristine H. Schmitz, Sue A. Shapses, Stephanie Shiau, Helmut Zarbl, Nancy E. Reichman, Emily S. Barrett, Martin J. Blaser, Daniel B. Horton

**Affiliations:** 1 Office of Organizational Leadership, Rutgers University, New Brunswick, NJ, USA; 2 Department of Pediatrics, Rutgers Robert Wood Johnson Medical School, New Brunswick, NJ, USA; 3 Center for Advanced Biotechnology and Medicine, Rutgers University, Piscataway, NJ, USA; 4 Department of Biostatistics and Epidemiology, Rutgers School of Public Health, Piscataway, NJ, USA; 5 Department of Obstetrics, Gynecology and Reproductive Sciences, Rutgers Robert Wood Johnson Medical School, New Brunswick, NJ, USA; 6 Department of Biochemistry and Microbiology, Rutgers University, New Brunswick, NJ, USA; 7 Department of Anthropology, Rutgers University, New Brunswick, NJ, USA; 8 Rutgers Institute for Translational Medicine and Science, New Brunswick, NJ, USA; 9 Department of Medicine, Rutgers Robert Wood Johnson Medical School, New Brunswick, NJ, USA; 10 Rutgers Robert Wood Johnson Medical School, Child Health Institute of New Jersey, New Brunswick, NJ, USA; 11 Rutgers School of Dental Medicine, Newark, NJ, USA; 12 Department of Environmental and Occupational Health and Justice, Rutgers School of Public Health, Piscataway, NJ, USA; 13 Environmental and Occupational Health Sciences Institute, Rutgers University, Piscataway, NJ, USA; 14 Department of Family Medicine and Community Health, Rutgers Robert Wood Johnson Medical School, New Brunswick, NJ, USA; 15 Department of Pediatrics, Boggs Center on Developmental Disabilities, Rutgers Robert Wood Johnson Medical School, New Brunswick, NJ, USA; 16 Children's Specialized Hospital, New Brunswick, NJ, USA; 17 Department of Kinesiology and Health, Rutgers University, New Brunswick, NJ, USA; 18 Nutrition and Health, New Jersey Institute for Food, New Brunswick, NJ, USA; 19 Department of Psychiatry, Rutgers Robert Wood Johnson Medical School, New Brunswick, NJ, USA; 20 Department of Nutritional Sciences, Rutgers University, New Brunswick, NJ, USA; 21 Department of Environmental and Occupational Medicine, Rutgers Robert Wood Johnson Medical School, Piscataway, NJ, USA; 22 Rutgers Center for Pharmacoepidemiology and Treatment Science, Health Care Policy and Aging Research, Institute for Health, New Brunswick, NJ, USA

**Keywords:** Mixed-methods, child health, leadership, team science, study design, transdisciplinary research, team effectiveness

## Abstract

**Introduction:**

The New Jersey Kids Study (NJKS) is a transdisciplinary statewide initiative to understand influences on child health, development, and disease. We conducted a mixed-methods study of project planning teams to investigate team effectiveness and relationships between team dynamics and quality of deliverables.

**Methods:**

Ten theme-based working groups (WGs) (e.g., Neurodevelopment, Nutrition) informed protocol development and submitted final reports. WG members (*n* = 79, 75%) completed questionnaires including de-identified demographic and professional information and a modified TeamSTEPPS Team Assessment Questionnaire (TAQ). Reviewers independently evaluated final reports using a standardized tool. We analyzed questionnaire results and final report assessments using linear regression and performed constant comparative qualitative analysis to identify central themes.

**Results:**

WG-level factors associated with greater team effectiveness included proportion of full professors (*β* = 31.24, 95% CI 27.65–34.82), team size (*β* = 0.81, 95% CI 0.70–0.92), and percent dedicated research effort (*β* = 0.11, 95% CI 0.09–0.13); age distribution (*β* = −2.67, 95% CI –3.00 to –2.38) and diversity of school affiliations (*β* = –33.32, 95% CI –36.84 to –29.80) were inversely associated with team effectiveness. No factors were associated with final report assessments. Perceptions of overall initiative leadership were associated with expressed enthusiasm for future NJKS participation. Qualitative analyses of final reports yielded four themes related to team science practices: organization and process, collaboration, task delegation, and decision-making patterns.

**Conclusions:**

We identified several correlates of team effectiveness in a team science initiative's early planning phase. Extra effort may be needed to bridge differences in team members' backgrounds to enhance the effectiveness of diverse teams. This work also highlights leadership as an important component in future investigator engagement.

## Introduction

There is growing recognition of the potential for team science to address complex, large-scale public health issues, such as obesity and COVID-19 [1–5]. The field of team science encompasses the empirical examination of “processes by which large and small scientific teams, research centers, and institutes organize, communicate, and conduct research.”[1] The Clinical and Translational Science Awards (CTSA) program from the National Center for Advancing Translational Sciences (NCATS) of the National Institutes of Health (NIH) fosters team science by engaging “all relevant expertise across disciplines, fields, and professions to produce research that advances translation” and integrating “concepts, theories, methods, technologies, and approaches from the range of disciplines, fields, and professions that can advance research goals.”[2] Although the value of team science has been demonstrated [6], more research is needed on how best to evaluate, cultivate, and promote team effectiveness. Furthermore, few publications have evaluated team effectiveness and productivity in the planning stages of large-scale team science initiatives. The scientific and organizational decisions made in these early stages can be critical for ensuring downstream success.

The New Jersey Kids Study (NJKS), an ambitious statewide initiative, aims to better understand the factors influencing childhood health, growth, development, and disease. The NJKS will enroll up to 5000 pregnant people across NJ and follow their children for at least 10 years. This research effort also seeks to establish a platform for high-impact transdisciplinary research. To design the NJKS and develop a protocol with optimized utility to address a range of research questions, the NJKS Executive Committee enlisted the collaborative, collective efforts of over 100 faculty, staff, and trainees from multiple schools and campuses across Rutgers University and Rutgers Health. Numerous academic and professional disciplines were included to ensure diverse types of expertise in study design. Individuals were organized into topical working groups (WGs) focused on major themes in maternal–child health, such as neurodevelopment and growth. WGs were tasked with identifying and prioritizing major knowledge gaps, research questions, survey instruments, tests, and research protocols to recommend for inclusion in the larger NJKS.

We used a mixed-methods approach to examine perceptions of WG members on team effectiveness and correlates of these perceptions in the planning stages of the NJKS initiative. By studying the process, perceived effectiveness, and deliverables of the WG teams working in parallel, we sought to determine the characteristics and practices that made WGs successful during the early planning of a comprehensive team science initiative. We had several *a priori* hypotheses:

1. WG leaders would rate team effectiveness more highly than other WG members, reflecting role biases.
2. More diverse groups (e.g., based on demographics, institutional membership) would rate team effectiveness more highly than less diverse groups.
3. Individuals who rated team effectiveness more highly would be more enthusiastic about future participation in NJKS activities and projects.
4. The quality of the final reports (as rated by independent examiners) would be higher in groups with higher average perceived team effectiveness.

The use of both qualitative and quantitative methods allowed for triangulation of data from both questionnaires and final reports to gain a more comprehensive understanding of the team science processes and practices involved in the planning of this initiative.

## Materials and methods

### Study design

WGs for the NJKS were convened from May 2022 through January 2023, organized around 10 themes (*Supplementary Methods* in Supplementary Material 1). Each WG had two co-leaders, selected by NJKS executive leaders based on relevant clinical and/or research expertise. Teams were asked to meet approximately monthly. A project staff member was present at every meeting to take notes, answer questions, and assist with team organization. In January 2023, the WGs submitted final reports that summarized their work and offered recommendations. While provided with a common set of objectives, organizational materials, and support staff, each of the groups developed its own approaches to organizing, planning, collaborating, and preparing final recommendations to the NJKS Executive Committee.

In May 2023, all WG members were invited to participate in a cross-sectional study of team dynamics. Consenting participants were asked to complete an online, de-identified REDCap questionnaire consisting of a modified TeamSTEPPS Team Assessment Questionnaire (TAQ) Version 2.0.[7] REDCap data collection tools were hosted by Rutgers Robert Wood Johnson Medical School. All questionnaires were completed by June 2023. In addition to these data, independent examiners conducted a quantitative evaluation of each WG's final report, and members of the research team conducted a qualitative analysis of the final reports, as detailed further in the following sections. Quantitative and qualitative analyses of the questionnaires and final reports were completed concurrently [8].

The study was approved by the Rutgers Institutional Review Board (Pro2023000609).

### Participant questionnaire

The TAQ was selected based on the relevance of the questions to the study objectives and the focus on both team dynamics and team leadership (*Supplemental Methods* in Supplementary Material 1). The TAQ was modified for this study, including removal of questions inapplicable to this research (e.g., questions related to customers, patient safety), and questions were revised as needed to tailor them to the work of the WGs (Supplementary Material 2). The questionnaire consisted of multiple sections corresponding to different team-related domains: Foundation, Functioning, Performance, Skills, Climate and Atmosphere, Identity, and Leadership. The section on Leadership was administered twice, with one section referring to WG leaders and the other section referring to the overall NJKS leadership (Executive Committee members). Each of eight sections was scored on a 5-point Likert scale, with a total score of 40 representing maximum team effectiveness. Those who belonged to multiple WGs (*n* = 4) were asked to complete the TAQ for each WG.

In addition to the TAQ, participants were asked to provide de-identified personal information about demographics, affiliation, academic level, distribution of work effort (e.g., % teaching, % research, etc.), history of research funding from the NIH, and level of enthusiasm for participating in future NJKS efforts (Supplementary Material 2).

### WG final reports and quantitative evaluations

The WG final report template was divided into multiple sections (*Supplemental Methods* in Supplementary Material 1). Each WG final report was independently evaluated by 11 graduate and postdoctoral trainees from several Rutgers University schools who had not been involved in the NJKS previously (*Supplemental Methods* in Supplementary Material 1). Reviewers were asked to score each final report based on the degree to which it met or exceeded specified requirements given to WGs (Supplementary Material 3). Reviewers assigned scores of 1–4 for each of five individual sections of the report and a 5-point summary score for overall quality, based on the extent to which the report and its recommendations would enable the NJKS to achieve its stated objectives. Thus, a maximum score of 25 for final reports would reflect optimal alignment with expectations and exceeding the stated requirements. Because of poor inter-rater reliability (intraclass correlation coefficient = 0.189), final report scores were normalized to the range of each reviewer (score-score_min_)/score_range_), yielding scores 0–1 that were used for modeling.

### Statistical analysis

The characteristics of study participants, TAQ scores, and final report evaluation scores were first summarized using descriptive statistics. Correlations among scores for each of the TAQ domains were evaluated using Spearman's correlation coefficients. TAQ scores were also compared between WG co-leaders and other members using Wilcoxon rank sum tests.

To evaluate the relationship between team characteristics and TAQ scores, linear regression modeling (weighted based on number of responses from each WG) was used with median overall scores regressed on aspects of team composition, diversity, and engagement: age distribution (standard deviation [SD]); ratio of WG members not identifying as non-Hispanic White to total WG member respondents (minority ratio); ratio of distinct school affiliations to total WG members (school diversity ratio); ratio of WG members previously funded by the NIH as principal investigators to total WG members (NIH PI ratio); ratio of full professors to total WG members (professor ratio); total number of registered WG members (regardless of survey response status); median percentage of WG meetings attended; and median percentage of WG members' professional effort committed to research.

Linear regression models were also fit to examine the (1) association between each individual TAQ domain score and respondents' expressed level of enthusiasm in future NJKS efforts, and (2) associations between TAQ total scores and final report scores, using WG-aggregated data.

In *post hoc* analyses, additional linear regression models weighted for the number of WG respondents were built to explore relationships between team-level factors and (1) median scores for the TAQ domain, “Team Climate and Atmosphere,” and (2) expressed level of enthusiasm in future NJKS efforts. Other exploratory analyses examined whether median final report scores were associated with median enthusiasm scores (all members or co-leaders) and WG co-leaders' median percent effort distribution (across research, clinical, education, and administration domains, totaling 100%).

To handle covariate collinearity within models, forward selection methods based on regression model fit diagnostics (adjusted *R* ^2^, Akaike information criterion, and log-likelihood calculations) were used to prioritize correlated variables to utilize in each final model.

All analyses were performed using R version 4.2.2 (Vienna, Austria). Two-sided *p*-values <0.05 were considered statistically significant, and no adjustments for multiple comparisons were made.

### Qualitative analysis

In addition to quantitative evaluations of the WGs' final reports, two authors (RAG and MW) conducted a qualitative analysis of the final reports. This analysis was performed to evaluate and compare the processes utilized by different teams in the conduct of WG activities. The analysis involved the development of a preliminary codebook organized around emerging themes. Codes were then refined and collapsed through the use of a constant comparative method to co-construct the final codebook [9]. Using the codebook as a guide, these two authors independently coded the final reports to identify emergent themes related to the practice and process of team science documented in the WG final reports. Periodic updates of the results were exchanged to ensure alignment of the findings and consistent use of coding categories.

## Results

Of 105 WG members, 83 (79%) consented to participate in the study, and 79 (75%) completed the questionnaire. Participants had a median age of 51 years (interquartile range [IQR] 41, 63.5), and 62% were female (Table 1). Three-quarters (76%) identified as White, 13% as Asian, 6% as Black, and 4% as Hispanic/Latino. Participants represented 14 schools or other units across multiple campuses, disciplines, and departments (Table 1). Of 27 departments represented, the Department of Pediatrics contributed the most WG members (24%). Participants were from several academic ranks (Full Professor was most common, 35%) and included four trainees (graduate/medical students or postdoctoral fellows). Professional effort of members varied, with research accounting for the highest median percentage of work effort (median 50%, IQR 11–70%), followed by education (median 17.5%, IQR 5–30%), administration (median 10%, IQR 5–24%), and clinical practice (median 0%, IQR 0–23%). Over half of participants (58%) had been funded by the NIH as PIs. Most WG members were either very enthusiastic (63%) or somewhat enthusiastic (27%) about remaining involved in future NJKS efforts.

**Table 1.** Characteristics of study participants

Characteristic	*n* (%)^a^ (*n* = 79)
Age (years), median [IQR]	51.0 [41.0, 63.5]
Gender	
Female	49 (62.0)
Male	28 (35.5)
Other	0 (0.0)
Unknown	2 (2.5)
Race	
White or Caucasian	60 (75.8)
Asian	10 (12.7)
Black or African American	5 (6.3)
Other/unknown	4 (5.1)
Ethnicity	
Not of Hispanic, Latino, or Spanish origin	73 (92.4)
Hispanic, Latino, or Spanish origin	3 (3.8)
Prefer not to answer	3 (3.8)
Institution	
Rutgers Biomedical and Health Sciences	57 (72.2)
Rutgers New Brunswick	16 (20.3)
Rutgers Camden or Newark	4 (5.1)
Non-Rutgers Institution	2 (2.5)
Current academic rank	
Professor	28 (35.4)
Associate Professor	23 (29.1)
Assistant Professor	20 (25.3)
Instructor	2 (2.5)
Post-doctoral fellow, student, or other trainee	4 (5.1)
Other	2 (2.5)
Professional effort, median percent [IQR]	
Research	50.0 [11.2, 70.0]
Education	17.5 [5.0, 30.0]
Administration	10.0 [5.0, 23.8]
Clinical	0 [0, 22.5]
NIH funding for research	
No	27 (34.2)
Yes, as PI	46 (58.2)
Yes, as Co-I	34 (43.0)
Unknown	1 (1.3)
Number of working groups participated in	
One group	75 (94.9)
Two groups	3 (3.8)
Three groups	1 (1.3)
Enthusiasm to continue	
Very unenthusiastic	1 (1.3)
Somewhat unenthusiastic	4 (5.1)
Neutral	1 (1.3)
Somewhat enthusiastic	21 (26.6)
Very enthusiastic	50 (63.3)
Unknown	2 (2.5)

Co-I = co-investigator; IQR = interquartile range; PI = principal investigator.

a Unless otherwise noted.

### Quantitative Findings

Although most participants were in one WG and completed one TAQ, four participated in and completed TAQs for two (*n* = 3) or three (*n* = 1) WGs. Across all participants, median domain scores ranged 4.0–4.5 out of 5 points; the median overall TAQ score was 28.8 of 40 (IQR 26.8–31.8) (Table 2). Median overall scores for individual WGs ranged from 24.6 to 32.6. Sections of the TAQ were generally strongly or very strongly correlated, except for the section on overall NJKS leadership (_range_ = 0.47–0.69) (*Supplementary Table 1* in Supplementary Material 1). TAQ scores of co-leaders and other members did not significantly differ, either overall or for any specific section (Table 3).

**Table 2.** TeamSTEPPS Team Assessment Questionnaire (TAQ) scores by domain

TAQ Domain	Median [IQR]
Team Foundation Score	4.0 [3.5, 4.5]
Team Functioning Score	4.2 [3.8, 4.8]
Team Performance Score	4.0 [3.7, 4.7]
Team Skills Score	4.0 [3.8, 4.8]
Team Climate and Atmosphere Score	4.2 [3.9, 4.8]
Team Identity Score	4.4 [4.0, 4.9]
Team Leadership: Co-Leaders Score	4.5 [4.0, 5.0]
Team Leadership: NJKS Leadership Score	4.2 [4.0, 5.0]
Overall TAQ Score	28.8 [26.8, 31.8]

IQR = interquartile range; NJKS = New Jersey Kids Study.

**Table 3.** Comparison of TeamSTEPPS Team Assessment Questionnaire (TAQ) scores between working group (WG) members and co-leaders

	WG Members		WG co-leaders		
**WG** ^b^	*n*	Median score [IQR]	*n*	Median score [IQR]	*P*-value^a^
All	67	28.8 [26.4, 32.1]	17	29 [27.7, 30.3]	0.80
1	12	29.7 [28.0, 30.8]	2	26.2 [24.9, 27.5]	0.25
2	10	27.7 [27.5, 32.2]	2	30.8 [29.2, 32.3]	0.51
3	9	28.7 [24.2, 31.7]	2	31.8 [30.4, 33.1]	0.20
4	5	28.1 [26.6, 31.5]	2	29.1 [28.7, 29.6]	0.86
5	5	30.7 [28.8, 32.6]	2	30.0 [29.3, 30.8]	0.86
6	6	32.8 [32.1, 34.3]	1	26.4 [26.4, 26.4]	0.29
7	7	26.4 [24.8, 27.9]	0	NA	NA
8	5	30.2 [28.8, 34.0]	2	31.0 [30.6, 31.5]	0.91
9	5	21.8 [20.4, 24.6]	2	26.4 [26.1, 26.6]	0.38
10	3	27.4 [26.6, 28.0]	2	30.0 [29.8, 30.2]	0.20

IQR = interquartile range; NA = not applicable; WG = working group.

a All p-values were calculated using Wilcoxon rank sum tests.

b Working groups sorted in decreasing order of number of participants.

In multivariable models, several factors were positively associated with TAQ scores, most strongly professor ratio (*β* = 31.24, 95% CI 27.65–34.82), along with team size (*β* = 0.81, 95% CI 0.70–0.92) and median percent effort in research (*β* = 0.11, 95% CI 0.09–0.13) (Table 4). Two factors were negatively associated with TAQ scores: school diversity ratio (*β* = −33.32, 95% CI −36.84 to −29.80) and age SD (*β* = −2.67, 95% CI −3.00 to −2.38). Team climate and atmosphere scores were positively associated with NIH PI ratio (*β* =1.02, 95% CI 0.52–1.52) and professor ratio (*β* = 0.94, 95% CI 0.42–1.46) and negatively associated with school diversity ratio (*β* = −2.52, 95% CI −3.10 to −1.93) (*Supplementary Table 2* in Supplementary Material 1).

**Table 4.** Associations between working group-level factors and modified TeamSTEPPS Team Assessment Questionnaire (TAQ) scores^a^

Factor	*β*	95% CI	*P*-value
Intercept	48.02	46.10, 49.95	<0.001
Standard deviation of age	−2.67	−2.97, −2.38	<0.001
School diversity ratio^b^	−33.32	−36.84, −29.80	<0.001
Number of working group members	0.81	0.70, 0.92	<0.001
Median % effort in research	0.11	0.09, 0.13	<0.001
Professor ratio^c^	31.24	27.65, 34.82	<0.001

β = adjusted beta coefficient; CI = confidence interval; PI = principal investigator unadjusted beta coefficient.

a Results produced from multivariable linear regression models with dependent variable TAQ score (n = 84).

b Ratio of number of distinct school affiliations to total working group members.

c Ratio of full professors to total working group members.

Among all sections of the TAQ, only scores regarding overall NJKS leadership were significantly associated with enthusiasm about continued NJKS involvement (*β* = 0.56, 95% CI 0.05–1.07) (Table 5). No team-level factors were significantly associated with expressed enthusiasm in future NJKS activities, although higher minority ratio corresponded to lower levels of expressed enthusiasm (*β* = −2.38, 95% CI −5.02 to 0.27).

**Table 5.** . Factors associated with enthusiasm scores^a^

	*β*	95% CI	*P*-value
Model #1 (*n* = 52): TAQ domains^b^			
Intercept	1.23	−0.45, 2.90	0.15
Team foundation	0.12	−0.45, 0.69	0.67
Team functioning	−0.11	−0.80, 0.58	0.75
Team performance	−0.18	−0.75, 0.39	0.52
Team skills	−0.18	−0.68, 0.33	0.48
Team climate	0.38	−0.25, 1.01	0.23
Team identity	0.71	−0.27, 1.69	0.15
WG co-leads	−0.55	−1.36, 0.26	0.18
NJKS leadership	0.56	0.05, 1.07	0.03
Model #2 (*n* = 77): WG-level factors			
Intercept	4.71	3.53, 5.88	<0.001
Standard deviation of age	−0.01	−0.18, 0.15	0.88
Minority ratio^c^	−2.38	−5.02, 0.27	0.08
Number of working group members	0.00	−0.08, 0.09	0.95
Median % effort in research	0.00	−0.01, 0.02	0.69
Professor ratio^d^	0.97	−1.45, 3.40	0.43

β = adjusted beta coefficient; CI = confidence interval; PI = principal investigator; WG = working group.

a Results produced from multivariable linear regression models with dependent variable the score from the question, “How enthusiastic are you to remain engaged in NJKS activities and projects in the future?.”

b Independent variables represent the domains from the modified TeamSTEPPS Team Assessment Questionnaire (TAQ).

c Ratio of WG members not identifying as non-Hispanic White to total WG members.

d Ratio of full professors to total working group members.

The median score for WGs' final reports was 22 (IQR 20–24) out of 25, with median scores of individual reports ranging 18 to 24. After normalization, median scores of the final reports were not associated with TAQ scores, the level of enthusiasm expressed by WG co-leaders or other members, or WG co-leaders' effort committed to research, clinical work, education, or administration (Table 6, *Supplementary Table 3* in Supplementary Material 1).

**Table 6.** Working group-level factors associated with final report assessment scores^a^

Factor	*n*	*β* coef.	95% CI	*P*-value
WG median TAQ score	10	0.028	−0.070, 0.127	0.528
WG member median enthusiasm score	10	−0.150	−0.676, 0.376	0.529
WG leader median enthusiasm score	9	−0.112	−0.541, 0.317	0.558
WG leader median % effort in research	9	−0.009	−0.019, 0.001	0.073
WG leader median % effort in clinical	9	0.005	−0.008, 0.018	0.430
WG leader median % effort in education	9	0.003	−0.018, 0.024	0.724
WG leader median % effort in admin	9	0.006	−0.009, 0.022	0.373

β = unadjusted beta coefficient; CI = confidence interval; TAQ = Team Assessment Questionnaire; WG = working group.

a Results produced from unadjusted linear regression models with the dependent variable of the final report assessment score normalized to the range of each reviewer ((score-score_min_)/score_range_).

### Qualitative Findings

The following central themes related to the practice and process of team science emerged as most salient from qualitative analysis of WG reports: (a) *organization and process*, (b) *collaboration*, (c) *delegation of tasks*, and (d) *decision-making patterns*. Each of these themes centers on team science practices that might be expected at the outset of any collective research endeavor. Table 7 includes corresponding definitions and illustrative quotes for each theme.

**Table 7.** Themes from qualitative analysis of working groups' final reports

Code	Description	Illustrative quotes (Working group)
Organization and process	Approaches to organizing working group priorities and materials, and developing a process to guide the activities of the working group	“To achieve these objectives the working group convened monthly meetings from June to December 2022 and encouraged electronic communication using email and Box in between meetings.” (Neurodevelopment) “We started by identifying the big questions that could be answered by this study, in a brainstorming session with our entire working group. From there, we relied on the expertise and recommendations of our working group members, and we recruited speakers when needed to talk about specific technologies.” (Genetics) “The working group spent a substantial amount of time discussing the alignment of specific measurements (e.g., food frequency questionnaire, nutrition questionnaires) with the unique diet of diverse population groups in New Jersey, with a specific focus on relevant blood biomarkers of nutritional status. A particular consideration was given to the balance between the quantity of growth, nutrition, and metabolism measurements (i.e., number of time points) vs. participant burden. The working group also discussed the importance of considering epidemiological trends and shifts in diet, nutrition transition (to ultra-processed foods), diet quality, that impact non-communicable diseases (e.g., obesity, cardiovascular disease). Specific research questions were brainstormed.” (Nutrition)
Collaboration	Approaches to engaging with internal and external partners to advance the goals of the working group	“As such, we discussed the interaction of physical activity, sleep and positive health for optimal maturation of the children. In this conversation, we recognized there is likely strong overlap and have had interactions with other working groups, including but not limited to: growth nutrition and metabolism (food intake/feeding behavior and body composition); neurology (neuromotor development); social determinants of health and resilience (much of positive health outcomes relate) as well as environmental exposures (biospecimens). This reinforced the complexity of the study as well as the potential strong cross-road disciplines working together to advance the health of citizens in New Jersey and potential elsewhere.” (Positive Health) “This is a great opportunity to engage our participants and offer information and education about genetic research. We strongly support connection with community organizations about the strengths and limitations of genetics. This could be accomplished via webinars, community group meetings, and “ask a geneticist” casual gatherings.” (Genetics)
Delegation of tasks	Approaches to dividing up responsibilities and action items to advance the priorities of the working group	“Each questionnaire was assigned to two group members for independent review in between meetings. Questionnaires and comments were subsequently reviewed by the group co-leaders, and key questionnaires that were pertinent to general pediatrics and not discussed in more depth by other groups were discussed during Working Group meetings. Reports were drafted by co-leaders and reviewed by other group members before submission.” (Pediatrics) “We primarily worked as a single unit and assigned experts in respective areas to review materials.” (Positive Health)
Decision-making patterns	Approaches to soliciting input and making decisions on behalf of the working group	“Informal discussions between the Neurodevelopment and Pediatric Working Groups led to the creation of the “Family Wellbeing” topic and included items such as parental mental health, ACE'S, stressors, and discrimination.” (SDOH) “If questions about specific measures or assessments arose, these were sent directly to members of the working group with relevant expertise.” (Nutrition)

ACEs = adverse childhood experiences; SDOH = social determinants of health.

With respect to *organization and process*, the WG reports highlighted a range of approaches for organizing their work. The specific content related to this theme included initial brainstorming of processes to be used by groups in performing core tasks, approaches to reviewing germane materials, and strategies for preparing meeting agendas and documenting groups' work. For example, as noted by the Obstetrics group, “Our group met…each time. We set agenda items for each meeting and then emailed our group specific things to review prior to the meeting. Then, during the meetings we solicited feedback in an open forum on those items.” Other groups discussed their strategy for using existing resources, instruments, and measurements that might inform their work. For example, the Pediatrics Working Group “found existing instruments from the NIH ECHO (Environmental influences on Child Health Outcomes) Study to be…comprehensive and valuable to the NJKS.” The Nutrition WG noted, “we critically evaluated relevant questionnaires and measurements done in the CHILD [Canadian Healthy Infant Longitudinal Development] study and ECHO study for potential use in the NJKS to measure growth, nutrition, and metabolism.” The dedicated project manager proved a useful support for many groups. As described by the Environmental Exposures WG, the project manager “assisted with agendas and took excellent meeting notes that she distributed promptly to group members after each meeting. All members then contributed to shared document tables to make recommendations with annotations which we discussed at subsequent meetings.”

Perspectives on *collaboration –* a core competency of team science – appeared most frequently in responses to one question in the report related to approaches to collaboration. Some WGs referred to collaboration in their interactions within the WG, while others referred to interactions across the NJKS project, NJ ACTS, Rutgers University, or the State of New Jersey. Qualitative data related to collaboration highlighted meaningful interactions cultivated within and across disciplines and institutions, and aspirations for collaboration that might help guide future NJKS activities. For example: “the interdisciplinary NJKS Neurodevelopment Working Group included investigators from diverse fields including economics, epidemiology, neuroscience, pediatrics, psychology, public health, speech language pathology. The working group engaged in a highly collaborative process that focused on achieving…four objectives.” Referring to plans for collaboration, the SDOH WG specifically identified interactions with the NJ ACTS Community Core, “which is composed of community members from various NJ individuals in communities and organizations in communities, which provide feedback & commentary on current research projects,” along with collaborations with the NJ Department of Health/Maternal and Child Health. The Genetics WG remarked, “We strongly support connection with community organizations about the strengths and limitations of genetics. This could be accomplished via webinars, community group meetings, and “ask a geneticist” casual gatherings.”

A third theme focused on *delegation of tasks* to advance the WGs' work. Similar to the theme of organization, the process of delegation included a range of approaches, including the following practices (not mutually exclusive): (a) delegation of specific tasks to WG members by co-leads; (b) completion of most tasks by co-leads followed by solicitation of input from the group; (c) completion of most tasks by WG members followed by solicitation of input from the group; (d) creation of subgroups to complete specific tasks within each WG; (e) delegation of tasks to individuals based on specific areas of expertise; and (f) delegation of tasks to volunteers within each WG. The Pediatrics WG, for example, described their approach to delegation of work: “Each questionnaire was assigned to two group members for independent review in between meetings. Questionnaires and comments were subsequently reviewed by the group co-leaders, and key questionnaires that were pertinent to general pediatrics and not discussed in more depth by other groups were discussed during WG meetings. Reports were drafted by co-leaders and reviewed by other group members before submission.” Using a different approach for task delegation, the Positive Health WG “primarily worked as a single unit and assigned experts in respective areas to review materials.”

The final theme referred to *approaches for engaging in group decision-making*. As documented within each report, the process of decision-making involved various tactics for soliciting recommendations among members of the group and strategies for building consensus on key decisions. Regarding group decision-making, leadership within each group remained fluid. Sometimes co-leaders played a central role in organizing the process, the distribution and delegation of work, and the approaches for reaching decisions; other times leadership was distributed across WG volunteers who took on additional tasks given their roles, expertise, and/or enthusiasm. The SDOH WG described, “Informal discussions between the Neurodevelopment and Pediatric Working Groups led to the creation of the “Family Wellbeing” topic and included items such as parental mental health, ACE's [adverse childhood experiences], stressors, and discrimination.” The Immunology WG noted, “We first identified immune-based pediatric diseases that were relevant for the kids of NJ. Keeping in mind the [planned] size of the study…the committee decided the focus should be allergic and atopic diseases including eczema, asthma, food allergy, and allergic rhinitis.” Finally, the Nutrition WG noted, “If questions about specific measures or assessments arose, these were sent directly to members of the working group with relevant expertise.”

## Discussion

In this mixed-methods study of team science processes and performance, we identified multiple team-level factors that were associated with greater perceived team effectiveness of WGs, including higher proportions of senior faculty members, larger WGs, and higher median levels of effort committed to research. Contrary to our hypotheses, metrics of team diversity, namely relative number of school affiliations and age distribution, were inversely associated with perceived team effectiveness. Furthermore, we did not find any differences in perceived team effectiveness between co-leaders and other members. Interestingly, perceptions of executive leadership, but not WG effectiveness, were associated with expressed enthusiasm for future participation in the initiative. We identified no single factor associated with the quality of the final reports submitted by WG teams.

High-impact biomedical research increasingly involves collaborative multidisciplinary teamwork. The complexities of engaging in collaborative work, coupled with different explanations and paradigms associated with team effectiveness, reinforce the unmet need to more fully understand critical factors associated with team effectiveness in clinical and translational science [10–12]. To assess team effectiveness, we used a modified version of the TAQ, one of various tools developed by TeamSTEPPS [7,13]. Prior studies using these tools have focused on healthcare applications, often to measure changes in team performance based on implementation of the TeamSTEPPS training program [14,15]. Our adaptation of the TAQ to measure team effectiveness in the NJKS appears to be a novel contribution to the team science literature, albeit one that makes it difficult to directly compare our findings to other team science-related investigations of team effectiveness. In a recent mixed-methods assessment of team effectiveness within a team science project, Slade et al observed very favorable responses among respondents to surveys regarding team satisfaction, team collaboration, team interactions, and attitudes regarding transdisciplinary research [16]. Mean responses in that study were modestly higher than median responses in many team-related domains in our study. However, our study differed from the prior in two important ways, besides the assessment tools used. First, our study focused on teams that worked together for just 8 months on initial study planning, whereas the prior study focused on teams that had been together for several years, applying for grants and conducting and publishing research together. Second, unlike the prior study, surveys in our study were de-identified, which may have led to less response bias. The use of de-identified surveys may also help explain the similar assessments between team co-leaders and other team members that we observed. Additionally, unlike the prior study, we assessed leadership as another important aspect of team functioning. Another prior study evaluating teams in the earlier stages of a team science initiative (the National Cancer Institute's Transdisciplinary Research on Energetics and Cancer (TREC) initiative) focused more on teams' and team members' orientation on the cross-disciplinary continuum (from unidisciplinary to transdisciplinary) and less on other aspects of team dynamics, functioning, and leadership, as in the current study [17,18].

Prior evaluations of effectiveness in team science have tracked team-based outcomes over time, such as the number of grants received or publications [19]. Slade and colleagues reported that the quality of team interactions was significantly associated with achievement of scholarly products, including the number of publications and grants submitted and awarded (*r* = 0.64) [16]. Such evaluations were not possible in our study of an initiative's planning stages. Another outcome measure for team effectiveness can involve peer review of teams' interim products, which can provide more standardized measures of team effectiveness and collaboration [19]. Interim evaluations can also help teams identify what is and is not working well and optimize subsequent activities. In the study of the early stage of the TREC initiative, peer review of proposals focused predominantly on the degree of cross-disciplinarity and aspects such as scope, analysis complexity, and number of experiment types proposed [17]. In our study, peer review of the WG final reports focused on their completeness and applicability to stated project goals. Although the scores of final reports varied across teams, we could not identify any factors associated with those scores, including perceived team effectiveness, WG member enthusiasm for continued project engagement, and WG co-leader enthusiasm or job profiles. Nonetheless, these scores may have been insensitive measures of team effectiveness for various reasons, including potentially outsized involvement of co-leaders in the writing of reports, the overall high levels of adherence to report specifications, limited inter-rater reliability, relative inexperience of evaluators, and limited sample size (*n* = 10 WGs). We suspect that inter-rater reliability may have been affected by the following factors: (1) the broad range of reviewers' disciplines; (2) rater bias; (3) subjective judgment in evaluating the quality of the reports; and (4) and lack of interaction among reviewers, such as meetings to discuss, align, and calibrate ratings. Certain reviewers tended to provide a narrower range of scores or consistently better scores compared to others, which contributed to the low inter-rater reliability. Analyses of both raw and normalized reviewer scores were similar.

Qualitative analyses of team members' reported experiences and teams' work products can enable complementary insights into team dynamics and effectiveness. The few studies that have included qualitative analyses of team products or outcomes have focused on large-scale team science initiatives [17,20]. In their mixed-methods study, Slade and colleagues interviewed selected team members to gain better understanding of team formation and interactions as well as strategies that promoted successful collaboration [16]. Our qualitative analyses of final reports drew upon the voices of WGs to highlight the teams' multiple approaches to organization, collaboration, communication, delegation, and decision-making. Observations from our study and others reinforce the value of adopting patterns and practices that best serve the goals, strengths, and expectations of team members. Furthermore, a mixed-methods approach can be useful for identifying key insights to inform subsequent phases of team science. For instance, as identified in the quantitative findings, the enthusiasm of team members for remaining involved in the NJKS seemed most strongly related to the perceived effectiveness of the project's overall executive leadership team. Meanwhile, the qualitative findings provided greater depth and detail regarding the varied approaches enacted by the WG leaders to facilitate the work of each team. As suggested by the qualitative results, those engaged in leadership within WGs played active roles in structuring meetings, organizing processes, facilitating conversations, and encouraging collaboration. These observations underscore the important roles that formal and informal leaders can play in motivating and engaging teams [20,21]. The triangulation of qualitative and quantitative findings provided us with a more comprehensive and richer understanding of the dynamics at play in this early phase of a team science initiative, with insights that might not have emerged with the use of only one methodologic approach.

Our analyses suggest that teams with more senior faculty and more experienced researchers tended to be more effective in study planning or at least were perceived as more effective by their members. Prior research on the science of team science has highlighted the professional opportunities that collaborative research offers to early-career faculty [16–22]. Our study did not specifically examine the perceptions or interactions of early-career faculty or trainees in the planning stages of this initiative. We also observed that larger teams tended to be rated as more effective, echoing prior findings on the productivity of larger teams [23].

In contrast to our expectations, we found that more diverse teams – as reflected by age distribution and school affiliation – tended to report lower perceived team effectiveness. The relationships between aspects of diversity and team effectiveness are complex, and interpretation of such findings should be made with caution. One potential explanation for our finding is that WGs whose members shared similar backgrounds were more effective in planning within the constraints of the limited frequency of meetings (monthly) and relatively short duration (8 months) that WGs worked together. While some have reported that more demographic diversity can be associated with greater creativity in engineering teams, diversity has also been negatively associated with team effectiveness when the team climate is not considered inclusive [24,25]. Others have reported that projects with greater disciplinary diversity tended to engender more satisfaction, but certain other elements of diversity may be negatively associated with perceived team effectiveness [22]. The authors noted, “For diverse groups of people to…[succeed], thoughtful group construction and support is required” [22]. In our study, the perceived team climate and atmosphere was positively associated with members' seniority and history of NIH funding but, as with overall TAQ scores, negatively associated with teams' diversity with respect to age and affiliation. Additionally, we found that respondents from more racially and ethnically diverse WGs tended to report lower levels of enthusiasm for future engagement in the NJKS. Having more members with leadership experience and skills could better facilitate a more inclusive and collaborative leadership approach, a concept positively associated with team climate [26,27]. In preparation for WG activities, co-leaders received training about the NJKS and WG objectives and expectations, though this training did not focus specifically on leadership or managing team diversity. Further investigation is warranted on the relationships between leadership, diversity, and team climate in research as well as the role of leadership training in the future success of multidisciplinary research teams.

This study has notable strengths, including the novelty of this investigation on team dynamics and effectiveness in the organizational stages of a large-scale project. We also used an adapted evidence-based questionnaire to assess team effectiveness as well as peer-reviewed evaluations of team deliverables using a standardized instrument.

This study's limitations included the small size of WGs, which limited the statistical power of certain analyses. For example, while every WG had two co-leaders, not every co-leader completed the study questionnaire, which limited comparisons between co-leaders and other members in some WGs. WGs were not particularly racially or ethnically diverse, potentially limiting our analyses on the impact of team diversity, though the racial and ethnic diversity of participants closely paralleled that of the larger university [28]. Moreover, self-reported data were subject to misclassification, although questionnaires were de-identified and participants' identities were protected throughout data collection and analysis. Selection bias is possible since not all WG members completed questionnaires, although a majority of WG members participated in the study. The vast majority of WG and study participants came from a single university (Rutgers), albeit a large state university spread across multiple campuses, schools, and departments, many of which were represented. Thus, our findings may not generalize to other team science settings. Additionally, several of the analyses were exploratory and warrant replication in future studies. Finally, the evaluations of final reports, completed by trainees using a custom-made instrument, had limited inter-rater reliability. Future efforts should enhance training and guidance to achieve more reliable evaluations.

In conclusion, teams often are dynamic and shaped by multiple internal and external factors. As team science becomes imperative for advancing high-impact, multi- and transdisciplinary research, the focus on assessing and evaluating team dynamics, processes, and effectiveness throughout the life cycle of a team science project remains a topic of applied and scholarly significance. In addition to providing recommendations that can enhance team effectiveness in future stages of the NJKS project, the findings from this study can guide the development of other emerging, complex team-based research projects and contribute substantively to the growing body of research on the science of team science. The central insights from this mixed-methods study reinforce the importance of pursuing the organization, leadership, and advancement of team science with greater intentionality; the relevance of organizing appropriate training and the setting of agreed upon expectations for team participant and team leader behaviors; and the value of exploring the perceived strengths, pressure points, and opportunities for growth during multiple phases of a team science project. Finally, the importance of leadership in shaping team members' enthusiasm for future engagement represents a key insight for team science research and practice.

## Supporting information

Gigliotti et al. supplementary material 1 Gigliotti et al. supplementary material

Gigliotti et al. supplementary material 2 Gigliotti et al. supplementary material

Gigliotti et al. supplementary material 3 Gigliotti et al. supplementary material

## Supplementary material

The supplementary material for this article can be found at https://doi.org/10.1017/cts.2024.578.
