# Supplementary material for: Assessments of working group effectiveness in the planning of the New Jersey Kids Study: An applied mixed-methods study on the science of team science
Source: J Clin Transl Sci. 2024 Oct 14;8(1):e163. doi: 10.1017/cts.2024.578 (PMC11604504; doi:10.1017/cts.2024.578)
Supplement: Gigliotti et al. supplementary material 1 — Gigliotti et al. supplementary material [file S2059866124005788sup001.docx]

***Supplemental Methods***

New Jersey Kids Study (NJKS) Working Groups (WGs):

NJKS WGs were organized around multiple themes: Obstetrics; Pediatrics; Asthma and Immunological Health; Neurodevelopment; Nutrition, Growth, and Metabolism; Oral Health; Environmental Exposures; Physical Activity, Sleep, and Positive Health; Social Determinants of Health; and Genetics. Prior to convening the WGs, WG co-leaders met with NJKS executive leaders and staff to review NJKS goals and expectations for WGs and co-leaders. Across the 8-month period, the NJKS executive leaders held multiple virtual town halls to engage with WG members.

Participant Questionnaire:

TeamSTEPPS is an evidence-based set of teamwork tools, aimed at optimizing patient outcomes by improving communication and teamwork skills among healthcare teams, including patients and family caregivers.^7^ Developed by the Department of Defense and the Agency for Healthcare Research and Quality to improve the quality, safety, and efficiency of health care, the TeamSTEPPS program includes both a formal training curriculum and several assessment tools, including the Team Assessment Questionnaire (TAQ) self-report.

WG Final Reports and Evaluators:

The WG final report template was divided into multiple sections, including an executive summary; summary of organizational and work processes; summary of meetings; key recommended scientific questions; recommendations of questionnaires, assessments, and specimens; and logistical and ethical considerations. Trainees evaluating the final WG reports represented a variety of disciplines (e.g., anthropology, child health, environmental health, nutrition, psychology) and were selected from a pool of candidates based on prior experiences with mixed methods research, program development, teaching, and/or other relevant areas, as well as their expressed interest in the project. All were compensated for their time.

Prior to completing any working group evaluation, each rater completed a mandatory orientation and training on the Rutgers Canvas Learning Management System. This 1-hour asynchronous online training consisted of an overview video that described the mission and vision of the New Jersey Kids Study as well as the role and tasks of the 10 working groups. The video also provided explicit instructions on how to conduct ratings for each working group through the use of a detailed evaluation rubric with an online submission link for the final ratings. The video and supplementary materials highlighted the importance of conducting independent evaluations where scores or impressions of the reports were not shared with other raters. Raters also completed a supplementary learning module on Canvas that included the NJKS External Reviewer How-To Guide, background information on the NJKS white paper, and a review of the NJKS white paper.

***Supplementary Table 1***. Correlations^a^ between different domains of the modified TeamSTEPPS Team Assessment Questionnaire

|  |  | **TAQ Score Domains** | | | | | | | | **TAQ Total Score** |
| --- | --- | --- | --- | --- | --- | --- | --- | --- | --- | --- |
|  |  | Team foundation | Team functioning | Team performance | Team skills | Team climate | Team identity | WG  co-leaders | NJKS leadership |  |
| **TAQ Score Domains** | Team foundation | 1.000 | 0.745 | 0.786 | 0.642 | 0.729 | 0.728 | 0.738 | 0.652 | 0.879 |
|  | Team functioning | 0.745 | 1.000 | 0.753 | 0.713 | 0.748 | 0.751 | 0.706 | 0.535 | 0.860 |
|  | Team performance | 0.786 | 0.753 | 1.000 | 0.653 | 0.722 | 0.733 | 0.707 | 0.662 | 0.881 |
|  | Team  skills | 0.642 | 0.713 | 0.653 | 1.000 | 0.677 | 0.718 | 0.644 | 0.471 | 0.794 |
|  | Team  climate | 0.729 | 0.748 | 0.722 | 0.677 | 1.000 | 0.789 | 0.802 | 0.543 | 0.871 |
|  | Team  identity | 0.728 | 0.751 | 0.733 | 0.718 | 0.789 | 1.000 | 0.905 | 0.689 | 0.910 |
|  | WG  co-leaders | 0.738 | 0.706 | 0.707 | 0.644 | 0.802 | 0.905 | 1.000 | 0.683 | 0.869 |
|  | NJKS leadership | 0.652 | 0.535 | 0.662 | 0.471 | 0.543 | 0.689 | 0.683 | 1.000 | 0.751 |
| **TAQ**  **Total Score** | | 0.879 | 0.860 | 0.881 | 0.794 | 0.871 | 0.910 | 0.869 | 0.751 | 1.000 |

NJKS, New Jersey Kids Study; TAQ, Team Assessment Questionnaire; WG, Working Group

^a^ Spearman correlation coefficients

***Supplementary Table 2.*** Factors associated with modified TeamSTEPPS Team Assessment Questionnaire (TAQ) Team Climate scores^a^

| Factor | β | 95% CI | P-value |
| --- | --- | --- | --- |
| Intercept | 5.14 | 4.65, 5.64 | <0.001 |
| Standard deviation of age | -0.05 | -0.10, -0.01 | 0.02 |
| School diversity ratio^b^ | -2.52 | -3.10, -1.93 | <0.001 |
| NIH PI ratio^c^ | 1.02 | 0.52, 1.52 | <0.001 |
| Number of working group members | -0.01 | -0.03, 0.01 | 0.45 |
| Professor ratio^d^ | 0.94 | 0.42, 1.46 | 0.001 |

β, adjusted beta coefficient; CI, confidence interval; PI, principal investigator

^a^ Results produced from multivariable linear regression models with dependent variable working group median Team Climate (TAQ domain) Score

^b^ Ratio of number of distinct school affiliations to total WG members

^c^ Ratio of WG members previously funded by the National Institutes of Health as principal investigators to all WG members

^d^ Ratio of full professors to total working group members

***Supplementary Table 3***. Working group-level factors associated with final report assessment scores^a^

| **Factor** | **n** | **β** | **95% CI** | **P-value** |
| --- | --- | --- | --- | --- |
| WG median TAQ score | 10 | 0.36 | -0.13, 0.84 | 0.19 |
| WG member median enthusiasm score | 10 | -0.38 | -3.27, 2.52 | 0.91 |
| WG leader median enthusiasm score | 9 | 0.16 | -1.55, 1.88 | 0.81 |
| WG leader median % effort in research | 9 | -0.02 | -0.07, 0.03 | 0.89 |
| WG leader median % effort in clinical | 9 | 0.003 | -0.05, 0.06 | 0.86 |
| WG leader median % effort in education | 9 | 0.006 | -0.08, 0.09 | 0.44 |
| WG leader median % effort in admin | 9 | 0.03 | -0.04, 0.09 | 0.46 |

β, unadjusted beta coefficient; CI, confidence interval; TAQ, Team Assessment Questionnaire; WG, Working Group

^a^ Results produced from unadjusted linear regression models with the dependent variable of the raw final report assessment score
