# Supplementary material for: Assessments of working group effectiveness in the planning of the New Jersey Kids Study: An applied mixed-methods study on the science of team science
Source: J Clin Transl Sci. 2024 Oct 14;8(1):e163. doi: 10.1017/cts.2024.578 (PMC11604504; doi:10.1017/cts.2024.578)
Supplement: Gigliotti et al. supplementary material 2 — Gigliotti et al. supplementary material [file S2059866124005788sup002.pdf]

NJKS Team Effectiveness Questionnaire

Please complete the entire survey below to the best of your ability.

As a reminder, after you submit your completed survey, you will be prompted to provide contact information in order to receive your Starbucks gift card. Further directions on how to do that will be provided after survey submission.

General Demographics

What is your age in years?

____

What is your gender?

◯ Woman
◯ Man
◯ Other (specify below)
◯ Prefer not to answer

Please specify your gender here:

____

What is your race?

(Select all that apply)

☐ American Indian or Alaska Native
☐ Asian
☐ Black or African American
☐ Native Hawaiian or Other Pacific Islander White
☐ White or Caucasian
☐ Some other race
☐ Prefer not to answer

Are you of Hispanic, Latino, or Spanish origin?

◯ No, not of Hispanic, Latino, or Spanish origin
◯ Yes, of Hispanic, Latino, or Spanish origin
◯ Prefer not to answer

Where are you primarily based?

◯ RBHS
◯ Rutgers Camden
◯ Rutgers New Brunswick
◯ Rutgers Newark
◯ Institution outside Rutgers

RBHS School (primary, if appointments in multiple schools)

◯ Ernest Mario School of Pharmacy
◯ New Jersey Medical School
◯ Robert Wood Johnson Medical School
◯ Rutgers School of Dental Medicine
◯ School of Graduate Studies
◯ School of Health Professions
◯ School of Nursing
◯ School of Public Health

Rutgers Camden School (primary, if appointments in multiple schools)

◯ School of Arts and Sciences
◯ School of Business
◯ School of Nursing
◯ Rutgers Law School

Rutgers New Brunswick School (primary, if appointments in multiple schools)

◯ Edward J. Bloustein School of Planning and Public Policy
◯ Graduate School of Applied and Professional Psychology
◯ Graduate School of Education
◯ Mason Gross School of the Arts
◯ Rutgers Business School - Newark & New Brunswick
◯ School of Arts and Sciences
◯ School of Communication and Information
◯ School of Engineering
◯ School of Environmental and Biological Sciences
◯ School of Graduate Studies
◯ School of Management and Labor Relations
◯ School of Social Work

Rutgers Newark School (primary, if appointments in multiple schools)

◯ School of Arts and Sciences
◯ School of Criminal Justice
◯ Rutgers Law School
◯ Rutgers Business School
◯ Graduate School - Newark
◯ School of Public Affairs and Administration

Non-Rutgers Institution (specify)

____

Department

____

Current Academic Rank

◯ Professor
◯ Associate Professor
◯ Assistant Professor
◯ Instructor
◯ Post-doctoral fellow
◯ Clinical fellow
◯ Clinical resident
◯ Student or other trainee (specify)
◯ Other (specify)

Student or other trainee title

____

Other title

____

Approximately what percentage of your professional effort is dedicated to...
A. Research ______
B. Clinical Work ______
C. Education ______
D. Administration ______
E. Other Areas ______
TOTAL = [pe_total] %

Note: Please write a value of 0 for any area listed above without any professional effort

---

Total Percentage

____

Please note that the Percent Effort values you have listed above add up to less than 100%. If that is accurate, feel free to leave it as is, but if not, please revise the values where appropriate.

Please note that the Percent Effort values you have listed above add up to more than 100%. If that is accurate, feel free to leave it as is, but if not, please revise the values where appropriate.

Has your research ever been funded by the National Institutes of Health (NIH)?

◯ Yes
◯ No

In what capacity did you receive NIH funding?

(check all that apply)

☐ As a Principal Investigator, Co-PI, or MPI
☐ As a Co-investigator

Which New Jersey Kids Study working group did you participate in?

(Note: If you were part of more than one group, you will have a chance to enter data on the additional group(s) later)

◯ Environmental Exposures
◯ Genetics
◯ Growth, Nutrition & Metabolism
◯ Immunological Health & Development
◯ Neurodevelopment
◯ Obstetrics
◯ Oral Health
◯ Pediatrics
◯ Positive Health, Physical Activity & Sleep
◯ Social Determinants of Health

NJKS Participation Details

Please answer the following questions about your participation in the [njks_group1] working group

Were you a member of the [njks_group1] working group or a co-leader?

◯ Member
◯ Co-leader

INSTRUCTIONS:
The following assessment is a statistical measurement of your impressions of team behavior as it relates to your indicated working group: [njks_group1]. Please answer all questions so that overall and domain-specific average scores may be calculated.

Team Foundation

	Strongly Agree	Agree	Undecided	Disagree	Strongly Disagree	NA/Prefer Not to Answer
The working group had a clear vision of what it was supposed to do.	◯	◯	◯	◯	◯	◯
The working group's activities were guided by a clear set of objectives and expectations.	◯	◯	◯	◯	◯	◯
The working group had adequate skills and member resources to achieve its goals.	◯	◯	◯	◯	◯	◯
Everyone in the working group had a clear and vital role.	◯	◯	◯	◯	◯	◯
The working group had adequate meeting time, space, and resources to achieve all objectives.	◯	◯	◯	◯	◯	◯
Working group meetings were well attended by all working group members.	◯	◯	◯	◯	◯	◯
The working group could measure its performance effectively.	◯	◯	◯	◯	◯	◯
The New Jersey Kids Study leadership had clear expectations for this working group.	◯	◯	◯	◯	◯	◯

Team Functioning

	Strongly Agree	Agree	Undecided	Disagree	Strongly Disagree	NA/Prefer Not to Answer
Working group meetings were run efficiently.	◯	◯	◯	◯	◯	◯
Everyone in the working group participated at an acceptable level.	◯	◯	◯	◯	◯	◯
This working group worked well together.	◯	◯	◯	◯	◯	◯
This working group worked well with other working groups in the project.	◯	◯	◯	◯	◯	◯
The goals and objectives of this working group will have a positive impact on the New Jersey Kids Study.	◯	◯	◯	◯	◯	◯

Team Performance

	Strongly Agree	Agree	Undecided	Disagree	Strongly Disagree	NA/Prefer Not to Answer
The working group used an effective short-term and long-term strategic planning process.	○	○	○	○	○	○
The working group met its project requirements.	○	○	○	○	○	○
The working group was productive.	○	○	○	○	○	○

Team Skills

	Strongly Agree	Agree	Undecided	Disagree	Strongly Disagree	NA/Prefer Not to Answer
The working group members communicated well with one another.	○	○	○	○	○	○
Constructive feedback was given by the working group members.	○	○	○	○	○	○
Working group members were familiar with each other's job responsibilities.	○	○	○	○	○	○
The working group members used effective decision making processes and problem solving skills.	○	○	○	○	○	○

Team Climate and Atmosphere

	Strongly Agree	Agree	Undecided	Disagree	Strongly Disagree	NA/Prefer Not to Answer
Working group members trusted each other.	○	○	○	○	○	○
Morale in this working group was high.	○	○	○	○	○	○
Working group members supported each other.	○	○	○	○	○	○
There were no feelings among working group members which might have pulled this working group apart.	○	○	○	○	○	○
The working group members resolved conflicts soon after they occured.	○	○	○	○	○	○
I felt free to express my opinions.	○	○	○	○	○	○
I had an influence on working group decisions.	○	○	○	○	○	○
Working group members showed consideration for needs and feelings of other working group members.	○	○	○	○	○	○
Working group members received recognition for individual performance.	○	○	○	○	○	○

Team Identity

	Strongly Agree	Agree	Undecided	Disagree	Strongly Disagree	NA/Prefer Not to Answer
I know why I was in the working group.	○	○	○	○	○	○
I was pleased to be in the working group.	○	○	○	○	○	○
The working group subscribed to a clear set of values.	○	○	○	○	○	○
This working group was fun to work with.	○	○	○	○	○	○
No individual, group, or gender dominated working group activities.	○	○	○	○	○	○
The working group had a positive self image.	○	○	○	○	○	○
I was a member of a working group in which the co-leaders promoted teamwork.	○	○	○	○	○	○

Team Leadership - Co-leaders

	Strongly Agree	Agree	Undecided	Disagree	Strongly Disagree	NA/Prefer Not to Answer
The working group co-leaders promoted participation by the working group members in key decisions.	○	○	○	○	○	○
The working group co-leaders shared responsibilities with working group members.	○	○	○	○	○	○
The working group co-leaders were effective leaders.	○	○	○	○	○	○
I shared my ideas/suggestions whether or not the working group co-leaders agreed with my input.	○	○	○	○	○	○
The working group co-leaders coached and supported individual working group members.	○	○	○	○	○	○
The working group co-leaders led by example.	○	○	○	○	○	○

Team Leadership - NJKS Leadership Team (Executive Committee)

Answer the following questions about interactions with the NJKS Leadership Team (Executive Committee), either through town hall meetings or within your working group. If you did not interact with members of the NJKS Leadership Team, select "NA/Prefer Not to Answer".

	Strongly Agree	Agree	Undecided	Disagree	Strongly Disagree	NA/Prefer Not to Answer
The NJKS leadership team members were effective leaders.	◯	◯	◯	◯	◯	◯
I shared my ideas/suggestions whether or not the NJKS leadership team members agreed with my input.	◯	◯	◯	◯	◯	◯
The NJKS leadership team members coached and supported individual working group members.	◯	◯	◯	◯	◯	◯
The NJKS leadership team members led by example.	◯	◯	◯	◯	◯	◯

How enthusiastic are you to remain engaged in NJKS activities and projects in the future?

◯ Very enthusiastic
◯ Somewhat enthusiastic
◯ Neutral
◯ Somewhat unenthusiastic
◯ Very unenthusiastic

Approximately what percentage of meetings for the [njks_group1] working group did you attend?

____

Please share any comments on your experience in the [njks_group1] working group or on your responses above.
