# Supplementary material for: Assessments of working group effectiveness in the planning of the New Jersey Kids Study: An applied mixed-methods study on the science of team science
Source: J Clin Transl Sci. 2024 Oct 14;8(1):e163. doi: 10.1017/cts.2024.578 (PMC11604504; doi:10.1017/cts.2024.578)
Supplement: Gigliotti et al. supplementary material 3 — Gigliotti et al. supplementary material [file S2059866124005788sup003.pdf]

NJKS Working Group Report Evaluation Rubric

RUTGERS
New Jersey Kids Study

Project Item	Report Exceeded Project Item Requirements – (4 points)	Report Successfully Met Project Item Requirements - (3 points)	Report Somewhat Met Project Item Requirements - (2 points)	Report Did Not Meet Project Item Requirements - (1 point)
Executive Summary:<br><br>**The Executive Summary provides an overview of the NJKS Working Group's main purpose and findings.**	The **Executive Summary** is clear and has **all** the following components:<br><br>• Clear overview of group's main purpose<br>• Summary of the main findings<br>• Major opportunities<br>• Challenges/limitations that were encountered<br>• Identification of potential ancillary studies<br>• Interactions with other working groups<br>• Pragmatic, actionable recommendations	The **Executive Summary** is clear and has **all** the following components:<br><br>• Clear overview of group's main purpose<br>• Summary of the main findings<br>• Major opportunities<br>• Challenges/limitations that were encountered<br>• Identification of potential ancillary studies<br>• Interactions with other working groups	The **Executive Summary** is missing **one or more** of the following components or lacks clarity:<br><br>• Challenges/limitations that were encountered<br>• Identification of potential ancillary studies<br>• Interactions with other working groups	The **Executive Summary** is missing **one or more** of the following components:<br><br>• Clear overview of the group's main purpose<br>• Summary of the main findings<br>• Major opportunities
Summary of Process:<br><br>**The Summary of Process section of the final report describes how the group was organized and how work was completed during and between meetings.**	The **Summary of Process** section contains **all** the following categories of information:<br><br>• How the working group was organized (by subtopic, pairing of team members to review specific	The **Summary of Process** section is missing **one** of the following categories of information:<br><br>• How the working group was organized (by subtopic, pairing of team members to review specific	The **Summary of Process** section is missing **two** of the following categories of information:<br><br>• How the working group was organized (by subtopic, pairing of team members to review specific	The **Summary of Process** section is missing **three** of the following categories of information:<br><br>• How the working group was organized (by subtopic, pairing of team members to review specific

RUTGERS
New Jersey Kids Study

Project Item	Report Exceeded Project Item Requirements – (4 points)	Report Successfully Met Project Item Requirements - (3 points)	Report Somewhat Met Project Item Requirements - (2 points)	Report Did Not Meet Project Item Requirements - (1 point)
	• questionnaires, literature review teams, etc.)<br>• How the work was completed during meetings<br>• How the work was completed between meetings	• questionnaires, literature review teams, etc.)<br>• How the work was completed during meetings<br>• How the work was completed between meetings	• questionnaires, literature review teams, etc.<br>• How the work was completed during meetings<br>• How the work was completed between meetings	• questionnaires, literature review teams, etc.)<br>• How the work was completed during meetings<br>• How the work was completed between meetings
Key Scientific Questions:<br><br>**The Key Scientific Questions section contains at least three research questions that the NJKS should investigate.**<br><br>**Each question has a 1-2 sentence description that highlights why the group believes the research question is significant for the NJKS.**<br><br>**Each question is numbered in order of priority (with 1 being the highest priority).**	The **Key Scientific Question** section of the report contains **five** or **more** research questions that the study should investigate.<br><br>**AND**<br><br>Each question is accompanied by a 1-2 sentence description that highlights its importance for the NJKS.<br><br>**AND**<br><br>Each question is numbered in order of priority (with 1 being the highest priority).	The **Key Scientific Question** section of the report contains **three or four** research questions that the study should investigate.<br><br>**AND**<br><br>Each question is accompanied by a 1-2 sentence description that highlights its importance for the NJKS.<br><br>**AND**<br><br>Each question is numbered in order of priority (with 1 being the highest priority).	The **Key Scientific Question** section of the report contains **two** research questions that the study should investigate.<br><br>**OR**<br><br>One to two questions are missing a 1-2 sentence description of importance for the NJKS, not including the top 3 questions.<br><br>**OR**<br><br>Questions are not numbered in order of priority.	The **Key Scientific Question** section of the report contains **one** research question that the study should investigate.<br><br>**OR**<br><br>Most or all questions are missing 1-2 sentence descriptions of importance for the NJKS, including the top 3 questions.

RUTGERS
New Jersey Kids Study

Project Item	Report Exceeded Project Item Requirements – (4 points)	Report Successfully Met Project Item Requirements - (3 points)	Report Somewhat Met Project Item Requirements - (2 points)	Report Did Not Meet Project Item Requirements - (1 point)
Group Recommendations<br><br>**The working group's recommendations for questionnaires, direct assessments and biospecimen collection are provided in a clear manner with related time points.**<br><br>**Attachments such as spreadsheets that were used to help organize the group's work are included.**	Each **recommendation** includes **all** the following components:<br>• Provided in an organized way<br>• Contains a description of the recommendation, including specific instrument names<br>• Includes the optimal time point of administration<br>• Highlights the anticipated amount of time for collection<br>• A prioritization level (high, medium or low);<br>• Anticipated cost<br>• Related notes and comments<br>• Recommendations appear feasible for implementation based upon assigned priorities & estimated times	Each **recommendation** includes **all** the following components:<br>• Provided in an organized way<br>• Contains a description of the recommendation, including specific instrument names<br>• Includes the optimal time point of administration<br>• Highlights the anticipated amount of time for collection<br>• A prioritization level (high, medium or low)<br>• Anticipated cost<br>• Related notes and comments	The **recommendations** are missing **one** to **two** components:<br>• Provided in an organized way<br>• Contains a description of the recommendation<br>• Includes the optimal time point of administration<br>• Highlights the anticipated amount of time for collection<br>• A prioritization level (high, medium or low)<br>• Anticipated cost<br>• Related notes and comments	The **recommendations** are missing **three** or **more** components:<br>• Provided in an organized way<br>• Contains a description of the recommendation<br>• Includes the optimal time point of administration<br>• Highlights the anticipated amount of time for collection<br>• A prioritization level (high, medium or low)<br>• Anticipated cost<br>• Related notes and comments

NJKS Working Group Report Evaluation Rubric

RUTGERS
New Jersey Kids Study

Project Item	Report Exceeded Project Item Requirements – (4 points)	Report Successfully Met Project Item Requirements - (3 points)	Report Somewhat Met Project Item Requirements - (2 points)	Report Did Not Meet Project Item Requirements - (1 point)
Logistical Considerations<br><br>**The final report provides a clear description of logistical considerations (such as anticipated challenges in study conduct or implementation, recruitment or retention concerns & ethical considerations) that are not highlighted within the *Group Recommendations* section.**	The final report provides a description of **five** or **more** logistical considerations that are not highlighted within the ***Group Recommendations*** section.<br><br>**AND**<br><br>The descriptions provided are clear and easily understandable.	The final report provides a description of **three or four** logistical considerations that are not highlighted within the ***Group Recommendations*** section.<br><br>**AND**<br><br>The descriptions provided are clear and easily understandable.	The final report provides a description of at least **one** or **two** logistical consideration(s) that are not highlighted within the ***Group Recommendations*** section.<br><br>**OR**<br><br>The descriptions are unclear and difficult to understand.	The final report **does not provide** a description of any logistical considerations.
Linkage to Data Sets<br>**No evaluation is needed here.**				
Alignment with NJ Population<br>**No evaluation is needed here.**				
Listing of Key Collaborators<br>**No evaluation is needed here.**				
Overall Quality of Report<br><br>**Total number of points for each category**	Enter total number of points here	Enter total number of points here	Enter total number of points here	Enter total number of points here

RUTGERS
New Jersey Kids Study

The overall objectives of the New Jersey Kids Study are:

- Understand the complex determinants of health that shape child development and the risk for chronic disease;
- Explore the role of the microbiome at different ages and stages in child development;
- Create the infrastructure for a comprehensive biobank that will serve as a platform for future research**.**

Please provide a rating of 1-5 for the following statement:

Evaluation Item	5 – (Completely Agree)	4 – (Somewhat Agree)	3 – (Neutral)	2 – (Somewhat Disagree)	1 – (Completely Disagree)
**Based on the scope of this working group, to what extent do you agree that this working group's final report and its recommendations will enable the NJKS to achieve the above stated objectives:**					

**Additional notes on responses to the above questions:**
